# Association of Genetic Variation in Calmodulin and Left Ventricular Mass in Full-Term Newborns

**DOI:** 10.1155/2013/410407

**Published:** 2013-11-05

**Authors:** Iwona Gorący, Jarosław Gorący, Karolina Skonieczna-Żydecka, Mariusz Kaczmarczyk, Grażyna Dawid, Andrzej Ciechanowicz

**Affiliations:** ^1^Department of Clinical and Molecular Biochemistry, Pomeranian Medical University, Ulical Powstańców Wielkopolskich 72, 71-111 Szczecin, Poland; ^2^Department of Cardiology, Pomeranian Medical University, Szczecin, Poland; ^3^Department of Pediatrics, Pomeranian Medical University, Szczecin, Poland

## Abstract

Calmodulin II (*CALM2*) gene polymorphism might be responsible for the variation in the left ventricular mass amongst healthy individuals. The aim was to evaluate the correlation between left ventricular mass (LVM) and g.474955027G>A (rs7565161) polymorphism adjacent to the *CALM2* gene. Healthy Polish newborns (*n* = 206) were recruited. Two-dimensional M-mode echocardiography was used to assess LVM. Polymorphisms were determined by polymerase chain reaction-restriction fragment length polymorphism and sequencing analyses. The carriers of the G allele of the *CALM2* polymorphism had significantly higher left ventricular mass/weight (LVM/BW) values, when compared with newborns homozygous for the A allele (3.1 g/m^2^ versus 2.5 g/m^2^, *P*_adjusted_ = 0.036). The AG genotype of *CALM2* was associated with the highest values of LVM/BW, exhibiting a pattern of overdominance (2.9 g/kg versus 3.1 g/kg versus 2.5 g/kg, *P*_adjusted_ = 0.037). The results of this study suggest that G>A *CALM2* polymorphism may account for subtle variation in LVM at birth.

## 1. Introduction

Left ventricular hypertrophy (LVH) and increased left ventricular mass (LVM) are strong risk factors for cardiovascular disease and morbidity [1]. Cardiac hypertrophy is characterized by increased cell size, cardiac remodeling of myofilaments, and increased expression of fetal genes [2]. LVM results from a complex of interaction between genetic, environmental, and lifestyle factors. Increased knowledge concerning genes involved in the modulation of LVM will lead to a better understanding of the etiopathogenesis of LVH.

Calcium (Ca^2+^) is arguably the most important messenger in cardiac muscle and plays a central role in regulating contractility, gene expression, hypertrophy, and apoptosis. It has been well described that Ca^2+^ transient movements regulate the transcription and gene expression that characterize the hypertrophic response of cardiomyocytes [2, 3]. The levels of Ca^2+^ are precisely controlled.

A major sensor and mediator of intracellular Ca^2+^ transient movements is calmodulin (CaM). The Ca^2+^CaM complex binds and activates enzymes, including protein kinases, protein phosphatases, phospholipases, nitric oxide synthases, and endonucleases. Three Ca^2+^ calmodulin dependent enzymes have significant roles in cardiac function: Ca^2+^ calmodulin-dependent protein kinase (CaMK), protein phosphatase 2B (calcineurin, CaN), and myosin light-chain kinase (MLCK). CaMK and CaN have been shown to play key and often synergistic roles in transcriptional regulation in cardiomyocytes [4]. It has been suggested that CaMK regulates gene expression via activation of several transcription factors [5, 6].

Ca^2+^-CaM-dependent kinase II (CaMKII), a major CaM target protein, is a uniquely regulated multifunctional regulatory enzyme. The CaMKII*δ* isoform is the predominant cardiac isoform [7, 8]. There are several studies indicating the major role of CaMKII involvement in cardiac hypertrophy and heart failure [9]. In hypertrophic myocardium of animal models, increased activity and expression of CaMKII have been shown [10, 11]. Experimental studies have demonstrated that transgenic mice overexpressing nuclear CaMKII*δ* have increased incidence of cardiac hypertrophy [12]. Inhibition of nuclear CAMKII activity causes transgenic mice to have smaller hearts than their nontransgenic littermates [8]. In addition, CaMKII is involved in apoptosis signaling. It has been shown that selective inhibitors of CaMKII significantly inhibit the apoptotic response [13]. Thus, any genetic variants that directly affect CaM gene expression or function are promising as candidates involved in modulating LVM. CaM is encoded by a multigene family consisting of three members: *CALM1*, *CALM2,* and *CALM3*. There are very few studies indicating the functional role of *CALM2* gene polymorphism. Mototani et al. [14] discovered that 2622A>G and 3001G>A polymorphism, both located in intron 1, may be associated with osteoarthritis in the Japanese population. Liu et al. [15] indicated that *CALM2 *is a candidate gene for primary open-angle glaucoma. To date, only Vasan et al. [16] have demonstrated, in meta-analysis, the correlation between *CALM2* polymorphism rs7565161 and echocardiographic diameter LVM in adults. The guanine to adenine transition at nucleotide position 474955027 (g.474955027 G>A, rs7565161) of human chromosome 2p21 is intergenic, adjacent to the *CALM2* gene. However, there are no reports which have focused on the association of intergenic adjacent *CALM2* polymorphisms with left ventricular mass in newborns. The factors influencing heart development during fetal life or first days of life, when external environmental factors such as diet, lifestyle, smoking or diseases have not yet had a marked impact, are still being sought. We hypothesize that adjacent intergenic *CALM2* polymorphism could potentially modify LVM during fetal life and in the first period of life in newborns. In the present study, the relationships between g.474955027 G>A (rs7565161) being adjacent intergenic *CALM2 *gene polymorphism and LVM in a population of Polish newborns have been investigated.

## 2. Materials and Methods

### 2.1. Study Subjects

The study was approved by the Pomeranian Medical University ethics committee. Study subjects were informed about the project and written consents were obtained.

The population included 206 consecutive healthy Polish newborns (92 females and 114 males), born after the end of the 37th week of gestation (from 37 to 40 weeks). Mothers in this study were healthy without any complications such as preeclampsia or eclampsia, and there was no fetal growth restriction. The scientists identifying the calmodulin genotypes were blinded to the clinical characteristics of subjects. Newborns in this study were appropriately grown for their gestational age (defined as birth mass above the 10th centile). Exclusion criteria were twins, intrauterine growth restriction, chromosomal aberrations and/or congenital malformations, or “small for gestational age,” that is, below the 10th centile body length (BL), birth weight (BW), or head circumference (HC). At birth, cord blood (500 *μ*L) of neonates was obtained for isolation of genomic DNA. The gender of the newborn, BL, BW, and HC were taken from standard hospital records. Body surface area (BSA) was calculated using the following equation [17]:

(1) $$\begin{array}{c} \text{BSA}=\sqrt{\left[\frac{\text{BL}\left(\text{cm}\right)\times \text{BW}\left(\text{kg}\right)}{3600}\right].} \end{array}$$

### 2.2. Blood Pressure Measurements

A diascope oscillometer (Artema) was used to determine systolic and diastolic blood pressure (SBP or DBP, resp.), and only one of the investigators performed all of the blood pressure (BP) measurements using a standardized protocol. The smallest cuff size that covered at least two thirds of the right upper arm and encompassed the entire arm was selected. BP was measured in a supine position on the 3rd day after delivery. Newborn measurements were taken at least one and a half hours following their last feeding or medical intervention. An appropriately sized cuff was applied to the right upper arm, and the newborn was then left undisturbed for at least 15 minutes or until the infant was sleeping or in a quiet awake state. Three successive BP recordings were taken at three-minute intervals.

### 2.3. Echocardiographic Measurements

Echocardiographic measurements in newborn on the 3rd day after delivery were made by one pediatric cardiologist. Two-dimensional M-mode echocardiography was performed using an Acuson Sequoia 512 unit (USA), equipped with a 2–4 MHz imaging transducer. Measurement techniques were consistent with the American Society of Echocardiography conventions. In a parasternal long-axis view, LVIDd-left ventricular internal diameter-diastolic, LVIDs-left ventricular internal diameter-systolic, LVPW-left ventricular posterior wall thickness at end diastole, IVS-thickness of interventricular septum at end diastole, LAD-left atrial diameter, AoD-aortic diameter, PAD-pulmonary artery diameter, LVV-left ventricular volume, and LVEF-left ventricular ejection fraction were measured (using M-mode formulas). The left ventricular masses (LVM) were calculated from the echocardiographic left ventricular dimension measurements, using the Penn convention with the equation modified by Huwez et al. [18] (1994) as follows:

(2) $$\begin{array}{c} \text{LVM}=1.04\left[{\left(\text{IVST}+\text{LVPWT}+\text{LVID}\right)}^{3}-{\text{LVID}}^{3}\right], \end{array}$$

where IVST, LVPWT, and LVID denote interventricular septal thickness, left ventricular posterior wall thickness, and left ventricular internal dimension, respectively. To accurately determine and standardize the left ventricular mass, the LVM was indexed with respect to body length (LVM/BL (g/m)), body weight (LVM/BW (g/kg)), and body surface area (LVM/BSA (g/m^2^)), respectively.

#### 2.3.1. Genetic Analysis

Genomic DNA from cord blood was isolated using the QIAamp Blood DNA Mini Kit (QIAGEN, Germany), according to the manufacturer's protocol. For the analysis of the intergenic G>A *CALM2* (rs7565161) polymorphism, a polymerase chain reaction-restriction fragment length polymorphism (PCR/RFLP) method was designed with the following primer pair: forward 5′-AgggCCTgCAATCTAAT-3′ and reverse 5′-ATATAATCCCCACCTTCAg-3′ (TIB MOL BIOL, Poznań, Poland). The *CALM *amplicons were subsequently digested with the *Aci*I restriction enzyme (MBI Fermentas, Vilnius, Lithuania). The PCR product of 417 base pairs (bp) was cut into fragments of 258 bp, 137 bp, and 22 bp in the presence of the G allele and into fragments of 395 bp and 22 bp in the presence of the A allele. Restriction fragments in each case were electrophoretically separated and visualized in midori green-stained (Nippon Genetics) 3% agarose gels. To verify the results, sequencing analyses were performed. All tested individuals had genotypes confirmed by sequencing. Each *CALM*2 amplicon was cleaned with GenElute PCR Clean-Up Kit (Sigma). Sequencing was performed according to the dideoxy Sanger method in a GeneAmp PCR System 9700 thermal cycler (Applied Biosystems), using BigDye Terminator v3.1 Cycle Sequencing Kit (Applied Biosystems). Afterwards, samples were purified (BigDye XTerminator Purification Kit, Applied Biosystems), and 20 *μ*L deionized formamide (Applied Biosystems) was added. Sequencing analysis using an ABI PRISM 3100-Avant machine (Applied Biosystems) was performed. The sequencing results were read using Sequencing Analysis Software v5.1 (Applied Biosystems). In each case, the result obtained with PCR-RFLP method was identical with that appropriate one from sequencing.

### 2.4. Statistical Analysis

The divergence of *CALM2* genotypes frequencies from Hardy-Weinberg equilibrium was assessed using *χ* ^2^ tests, and the distribution of each quantitative variable was tested for skewness. Quantitative data were presented as means ± SD and analyzed either by Student's *t*-test or by one-way ANOVA. Left ventricular mass indexes (LVMIs) were tested for association with genotype using multivariate analysis (ANCOVA) in order to adjust for possible confounding factors: neonatal (gestational age, gender, SBP, and APGAR at three minutes) and maternal (age, BMI at the beginning and the end of the pregnancy, smoking status, and hypertension status). Dominant, recessive, and additive modes of inheritance were tested. Statistical significance was defined as *P* < 0.05. All data were analyzed with STATISTICA (data analysis software system, version 10.0, StatSoft, Inc. 2011, http://www.statsoft.com/).

## 3. Results

Characteristics of the newborn cohort (*n* = 206) are shown in Table 1. The distribution of these characteristics in our cohort approached normality (skewness < 2 for all variables). Mean BW and BSA values in boy newborns were significantly higher than those in girls. SBP measurements were also higher than those in girls. 69 GG *CALM2 *homozygotes (33.5%), 95 GA heterozygotes (46.1%), and 42 AA homozygotes (20.4%) were identified. There were no significant differences in *CALM2* genotype or allele distributions between boys and girls (*P* = 0.273, and *P* = 0.107, resp.). The *CALM* genotype distributions conformed to the expected Hardy-Weinberg equilibrium (*P* = 0.396).

**Figure 1:**
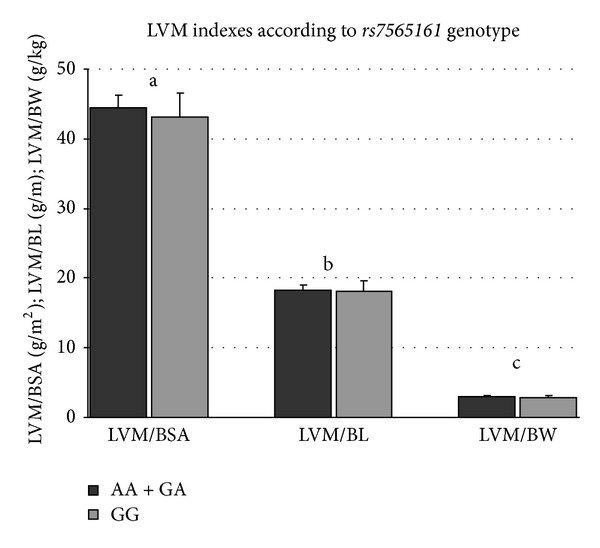
LVM indexes according to *rs7565161 *genotype. Mean and SEM are shown. ^a^ *P* = 0.574; ^b^ *P* = 0.795; ^c^ *P* = 0.404; ^a,b,c^AA + GA versus GG.

**Figure 2:**
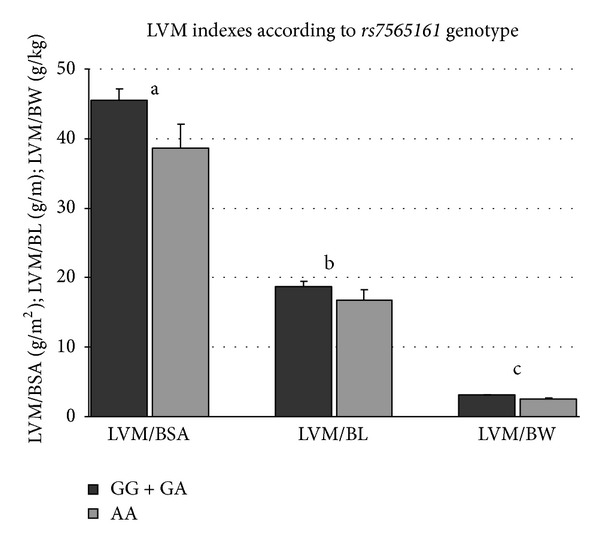
LVM indexes according to *rs7565161* genotype. Mean and SEM are shown. ^a^ *P* = 0.075; ^b^ *P* = 0.172; ^c^ *P* = 0.036; ^a,b,c^GG + GA versus AA.

**Figure 3:**
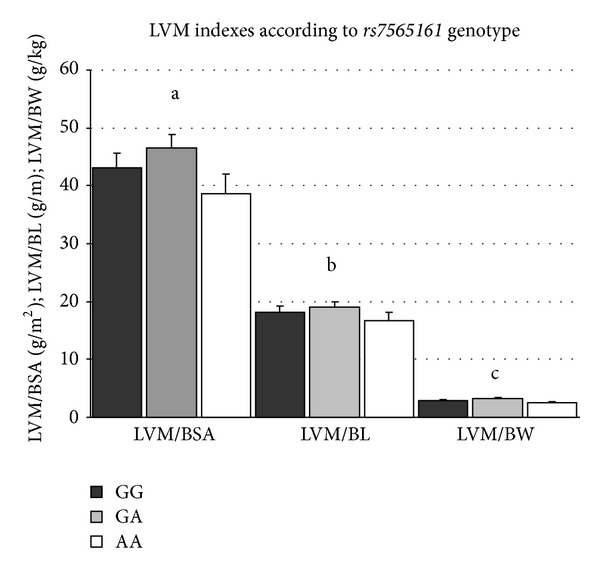
LVM indexes according to *rs7565161* genotype. Mean and SEM are shown. ^a^ *P* = 0.109; ^b^ *P* = 0.306; ^c^ *P* = 0.037; ^a,b,c^GG versus GA versus AA.

LVMI measurements were tested for association using multivariate analysis (ANCOVA) in order to adjust for possible confounding factors, after adjusting for newborn (gestational age, gender, SBP, and APGAR at three minutes) and maternal (age, BMI at the beginning and the end of the pregnancy, smoking status, and hypertension status) parameters. We revealed a significant association between LVMIs (LVM/BW in recessive and additive modes and the* CALM2 *polymorphism). The carriers of the G allele of the *CALM2 *polymorphism had significantly higher LVM/BW values, when compared with newborns homozygous for the A allele (3.1 g/m^2^ versus 2.5 g/m^2^, *P*_adjusted_ = 0.036, resp.). The AG genotype of *CALM2* was associated with the highest values of LVM/BW, exhibiting a pattern of heterozygote advantage (2.9 g/kg versus 3.1 g/kg versus 2.5 g/kg, *P*_adjusted_ = 0.037) (Figures 2 and 3). Carriers of the A allele did not differ in LVM indexes (Figure 1).

An association was observed between genotype and DBP ≥ 90 percentile (*P* = 0.027). Carriers of the allele A of the *CALM2* gene had an increased incidence (%) SBP ≥ 90 percentile (*P* = 0.027, 76.2% versus 23.8%). Lastly, the *CALM2 *polymorphism was significantly correlated with maternal history of gestational age (*P* = 0.019). An overview over the data can be found in Table 2.

## 4. Discussion

Genetic factors are estimated to be responsible for between 30% and 70% of cardiac mass variance [19]. Studies in twins [20, 21] and populations [22, 23] showed that LVM is under genetic control.

The present study in a cohort of newborns has demonstrated for the first time the significant association between variants of the intergenic adjacent *CALM2*  polymorphism and increases in LVM indices in newborns. Proper assessment of heart size in the newborn still stirs controversy. Therefore, to minimize the disparities, we carefully selected homogenous group of full-term newborns. To accurately determine LVM, we used LVM in relation to BSA, BL, and BW, which are reported to be more appropriate. It should be emphasized that confounding factors such as especially gestational age may play a role in the development of LVM in fetus. The fetal programming hypothesis states that, for example, birth mass in newborns may be partially related to maternal factors [24]. In this study, the AG genotype of intergenic adjacent *CALM2 *polymorphism was associated with the subtle higher values of LVMI, exhibiting a pattern of heterozygote advantage in results. What is important, in our study, the carriers of the G allele have higher LVM than the carriers of the A allele. These results were similar to those of a large cohort of adults, who were studied by Vasan et al. [16]. In this meta-analysis of echocardiographic data associated with interindividual variation in cardiac dimension, the polymorphism of *CALM2* gene rs7565161 was associated with LVM. It should be mentioned that total sample included those with coronary heart disease, peripheral vascular disease, valvular heart disease, stroke, and circulation heart failure. These risk factors may also increase the effect of the gene.

Current results exhibit a pattern of heterozygote advantage, as heterozygote newborns had significantly higher LVMI than the carriers of homozygote genotypes. The heterozygote advantage hypothesis attributes heterosis to the superior fitness of heterozygous genotypes over homozygous genotypes at a single locus [25]. Some studies suggest that heterozygote advantage is a favorable process, the positive selection over evolution, as a natural consequence of adaptation role of variation in gene [26–28]. However, in light of Vasan's study [16], the feature that may be potentially beneficial in early life may lead to predisposition to increase or hypertrophy left ventricular in adults. Williams suggested “antagonistic pleiotropy” theory, which assumes that some genes responsible for increased fitness in the children, fertile organism contribute to decreased fitness in adults [29]. We conclude that this theory may be relevant here. We hypothesized that genetic variation in the intergenic adjacent *CALM2* gene polymorphism, analogously to the other common polymorphisms in developmental genes, may cause minor changes in the development or modulation of LVM in newborns. 

We continue observing our population and consider conducting follow-up, which will show in later years whether the heterozygotes have a predisposition to develop left ventricular hypertrophy or not. However, our results require confirmation in further independent large studies.

The connection between calmodulin and modulating cardiac contractile function and growth is well documented [30, 31]. Otherwise, in an experimental animal study, the protein level of CaM was shown with a relatively high level of calmodulin appearing on gestational days 14-15, followed by a steady but significant decrease at birth and during the first week of postnatal life [32]. It is reported that specific elevation of CaM levels directly affects the rate of cell proliferation [33]. Also, Gillett et al. [34], in animal study (fetal sheep), showed that increased CALM2 mRNA expression levels may reflect an important role for calmodulin in expansion-induced fetal lung growth. A study performed in human showed that genes encoding calmodulin (*CALM1*, *CALM2,* and *CALM3*) are involved in increasing proliferation [35, 36].

Although such knowledge indicates the important role of calmodulin-dependent protein kinases and phosphatases in regulating cardiac hypertrophy [4], the role of genetic variation in CaM in the physiology of the development human heart has not been clarified. Our results suggest that genetic variation of *CALM2* may be partly involved in regulating myocardial cell proliferation and growth, during embryogenesis and in the first days of life. It is possible that genetic variation in CaM may have been involved in regulating the activity or/and levels in serum kinases and phosphorylases (e.g., CaMKII, calcineurin) during fetal life. In the current study, we investigated healthy newborns born at full term. Our previous studies reported that RAS (renin-angiotensin system) or *BMP4 *(bone morphogenetic protein 4) and *BMPR1A *(bone morphogenetic protein, receptor type 1A) genetic variation may partially account for subtle variation in LVM or parameters or heart parameters in newborns [37, 38]. To the best of our knowledge, the recent results have never been replicated, and therefore the replication of the study findings in different population is needed.

Additionally, an association between *CALM2* polymorphism, and DBP and MAP was found, but the mechanism by which this might act is not clear. Blood pressure is regulated by multiple neuronal, hormonal, renal, and vascular control mechanisms, as well as genetic and environmental factors. It is also dependent, inter alia, on the force of contraction of the heart muscle which is connected indirectly to the left ventricular mass. There are many known candidate genes that have huge influence on the blood pressure or development of hypertension [39–41]. However, the mechanisms of interaction intensifying effects of these genes are still researched. It is known that changes in signaling mechanisms in the endothelium of vascular smooth muscle (VSM) cause alterations in vascular tone and blood vessel remodeling and may lead to persistent increase in vascular resistance. Vascular tone that is a component of regulating blood pressure can be controlled indirectly by different genes activity. It is known that CaM regulates various proteins. An experimental study demonstrated findings that expression levels of several CaM-related proteins are changed in vascular tissues and suggested that CaM-related proteins might be at least in part related to the pathogenesis of hypertensive vascular diseases [42]. A recent study reported that CaMKII inhibitor inhibited the Ang II-induced vascular smooth muscle cell hypertrophy [43]. However, the role of CaM-related protein in vascular pathophysiology is not yet fully clarified. Further studies are necessary to clarify it.

In conclusion, we have shown that the intergenic adjacent *CALM2* polymorphism is associated with left ventricular mass in newborns. This might be the consequences of variation in cell proliferation and growth, and this finding may indicate an important role for genetic variation of *CALM2* in expansion-induced heart growth in fetal life.

## Figures and Tables

**Table 1:** Clinical and echocardiographic characteristics of the newborns in regard to gender.

	Total	Males	Females	*P *
*n* (%) *n* = 206	206	114 (55.3%)	92 (44.7%)	
BL (m)	0.6 ± 0.0	0.56 ± 0.0	0.55 ± 0.0	0.056
BW (kg)	3.6 ± 0.5	3.6 ± 0.5	3.4 ± 0.7	0.032
BSA (m^2^)	0.2 ± 0.0	0.2 ± 0.0	0.2 ± 0.0	0.028
SBP (mmHg)	69.6 ± 9.0	69.2 ± 9.9	68.9 ± 7.7	0.024
DBP (mmHg)	40.0 ± 7.8	40.0 ± 8.1	40.2 ± 7.6	0.509
MAP (mmHg)	51.4 ± 7.6	51.8 ± 8.0	52.0 ± 7.2	0.355
LVDd (mm)	18.6 ± 1.6	18.7 ± 1.7	18.5 ± 1.6	0.566
LVDs (mm)	11.6 ± 1.4	12.0 ± 1.3	11.7 ± 1.4	0.480
IVS (mm)	3.76 ± 0.7	3.7 ± 0.7	3.8 ± 0.6	0.278
LVPW (mm)	2.8 ± 0.7	2.7 ± 0.7	2.8 ± 0.7	0.861
LVM (g)^‡^	9.9 ± 2.8	43.1 ± 11.4	42.2 ± 11.6	0.851
LVV (mL)^‡^	10.7 ± 2.5	10.8 ± 2.5	10.5 ± 2.4	0.633
LVM/BL (g/m)^‡^	17.7 ± 4.8	18.0 ± 4.8	17.3 ± 4.9	0.840
LVM/BW (g/kg)^‡^	2.96 ± 0.8	2.9 ± 0.8	2.9 ± 0.8	0.937
LVM/BSA (g/m^2^)^‡^	42.76 ± 11.5	43.1 ± 11.4	42.2 ± 11.6	0.851

^‡^Adjusted for SBP and DPB.

MAP: mean arterial pressure.

**Table 2:** Overview of results depending on fetal genotypes.

	GG *n* = 69	GA *n* = 95	AA *n* = 42	*P *
Gender M/F	42/47 (37%/29%)	53/42 (46%/46%)	19/23 (17%/25%)	0.273

Gestational age (weeks)	39.3 ± 0.9	39.5 ± 1.0	39.0 ± 1.0	0.019
Birth weight (kg)	3.48 ± 0.46	3.5 ± 0.43	3.4 ± 0.47	0.503
Neonatal body length (cm)	0.56 ± 0.03	0.56 ± 0.03	0.55 ± 0.03	0.384
Neonatal head circumference (cm)	33.9 ± 1.5	33.7 ± 1.3	34.1 ± 1.3	0.633
Apgar 3 min	9.7 ± 0.9	9.6 ± 0.8	9.5 ± 1.3	0.734
SBP (mmHg )	68.7 ± 8.0	68.8 ± 9.1	70.4 ± 10.4	0.574
SBP ≥ 90 percentile *n*, (%)	4 (40,0)	9 (45,0)	7 (35,0)	0.173
DBP (mmHg)	39.5 ± 7.4	40.0 ± 7.4	41.0 ± 9.5	0.634
DBP ≥ 90 percentile	5 (23.8)	7 (33.3)	9 (42.9)	0.027
MAP (mmHg)	51.5 ± 7.1	52.0 ± 6.9	52.4 ± 9.9	0.830
MAP ≥ 90 percentile	6 (27.27)	9 (40.91)	7 (31.82)	0.373
Maternal age (years)	28.2 ± 6.0	27.7 ± 5.2	29.3 ± 4.5	0.274
Smoking habits History during pregnancy *n*, (%)	7 (33.33)	11 (52.38)	3 (14.29)	0.731
Hypertension History during pregnancy or history of hypertension *n*, (%)	3 (17.65)	8 (47.06)	6 (35.29)	0.182
BMI (kg/m^2^) at the beginning of pregnancy	21.7 ± 2.9	22.2 ± 3.6	22.9 ± 4.7	0.251
BMI (kg/m^2^) at the end of pregnancy	27.3 ± 3.6	28.0 ± 4.0	27.7 ± 4.6	0.539
Parity	1.6 ± 08	1.4 ± 0.8	1.6 ± 0.9	0.292
